# The Relevance of the Bacterial Microbiome, Archaeome and Mycobiome in Pediatric Asthma and Respiratory Disorders

**DOI:** 10.3390/cells11081287

**Published:** 2022-04-10

**Authors:** Carolin Baehren, Eleni Buedding, Aliyah Bellm, Frank Schult, Anton Pembaur, Stefan Wirth, Anja Ehrhardt, Friedrich Paulsen, Jan Postberg, Malik Aydin

**Affiliations:** 1 Laboratory of Experimental Pediatric Pneumology and Allergology, Center for Biomedical Education and Research, School of Life Sciences (ZBAF), Faculty of Health, Witten/Herdecke University, 58455 Witten, Germany; carolin.baehren@uni-wh.de (C.B.); eleni.buedding@uni-wh.de (E.B.); 2 Helios Hospital Krefeld, Children’s Hospital, Teaching Hospital of RTWH University Hospital Aachen, 47805 Krefeld, Germany; aliyah.bellm@helios-gesundheit.de; 3 Center for Child and Adolescent Medicine, Center for Clinical and Translational Research (CCTR), Helios University Hospital Wuppertal, Witten/Herdecke University, 42283 Wuppertal, Germany; frank.schult@uni-due.de (F.S.); stefan.wirth@uni-wh.de (S.W.); 4 Clinical Molecular Genetics and Epigenetics, Faculty of Health, Center for Biomedical Education & Research (ZBAF), Helios University Hospital Wuppertal, Witten/Herdecke University, Alfred-Herrhausen-Str. 50, 58448 Witten, Germany; anton.pembaur@uni-wh.de (A.P.); jan.postberg@uni-wh.de (J.P.); 5 Institute of Virology and Microbiology, Center for Biomedical Education and Research (ZBAF), Department of Human Medicine, Faculty of Health, Witten/Herdecke University, 58453 Witten, Germany; anja.ehrhardt@uni-wh.de; 6 Institute of Functional and Clinical Anatomy, Friedrich Alexander University Erlangen-Nürnberg, 91054 Erlangen, Germany; friedrich.paulsen@fau.de

**Keywords:** microbiome, archaea, fungi, nasopharynx, oropharynx, asthma, allergy, respiratory tract infections

## Abstract

Bacteria, as well as eukaryotes, principally fungi, of the upper respiratory tract play key roles in the etiopathogenesis of respiratory diseases, whereas the potential role of archaea remains poorly understood. In this review, we discuss the contribution of all three domains of cellular life to human naso- and oropharyngeal microbiomes, i.e., bacterial microbiota, eukaryotes (mostly fungi), as well as the archaeome and their relation to respiratory and atopic disorders in infancy and adolescence. With this review, we aim to summarize state-of-the-art contributions to the field published in the last decade. In particular, we intend to build bridges between basic and clinical science.

## 1. Introduction

Large-scale metagenomic approaches to the study of the human microbiome, particularly but not exclusively that of the digestive tract, demonstrate the genetic diversity and complexity of the human microbiome, but also its longitudinal dynamics in ontogeny and its responsiveness to environmental influences with respect to different body sites [1,2,3]. For the airways, studies have revealed that microbiota dysbiosis is associated with pathogen invasion, which may consequently lead to respiratory disease [4,5,6,7]. Thus, it is necessary to understand how the respiratory tract becomes a place for (pathogenic) microbial colonization, and how this causes infection or even influences the pathogenesis of several diseases [8,9]. With this review, we aim to summarize important works published in the last decade. We present a brief overview of the anatomy and physiology of the upper respiratory tract, followed by a summary of studies on the roles of bacterial, archaeal and fungal colonization. Importantly, we provide a brief update on current views on the evolutionary history of cellular life to provoke the question as to whether we systemically overlook multitudes of commensal or parasitic members of our microbiota communities. 

## 2. A Brief Evolutionary History of the Domains of Cellular Life

THE MICROBE is so very small
You cannot make him out at all,
But many sanguine people hope
To see him through a microscope.
His jointed tongue that lies beneath
A hundred curious rows of teeth;
His seven tufted tails with lots
Of lovely pink and purple spots,
On each of which a pattern stands,
Composed of forty separate bands;
His eyebrows of a tender green;
All these have never yet been seen-
But Scientists, who ought to know,
Assure us that is must be so...
Oh! let us never, never doubt
What nobody is sure about!
‘The Microbe’ from More Beasts for Worse Children.
Hilaire Belloc. Duckworth, 1897 [10].

Surely, it is an overstatement to say that when talking about microbes over a century after Hilaire Belloc published his poem ‘The Microbe’, *‘nobody is sure about’* the nature of microbiotic life. However, since evolutionary biologists drastically reshaped the tree of life in recent decades, many of us need to revise our time-honored assumptions of the evolutionary history of cellular life with potential consequences for our views on human microbiota. Understandably, in medicine, we are interested in pathogens, which within the microbiota seem to belong primarily to the domain of bacteria and the eukaryotic kingdom of fungi but include relatively few other eukaryotic parasites. Conclusively, we tend to neglect an unknown multitude of organisms that we probably fail to recognize in part because we do not look closely, mainly due to technical limitations: the sequencing of specific target amplicons 16S/18S relies on the use of defined ‘universal’ or taxon-specific primer sets, which can be useful for many species, but surely are inherently not universal. Shotgun metagenomic sequencing, on the other hand, virtually provides information on the total genomic DNA from all organisms in a sample, but identification depends on matching with reference genomes. Here, the identification of less characterized and annotated microbiota can be extremely challenging or even impossible. Mora and colleagues estimated a portion of more than 85% unrecognized species on Earth alone for eukaryotes and an estimated number of 8.7 million extant species [11]. 

A study on Earth’s bacterial and archaeal diversity suggests approximately 2 million operational taxonomic units (OTUs), a number that is controversially debated [12]. The main argument that this could be a vastly underestimated number is that most bacterial and archaeal species on Earth have been incorrectly considered, since they live in the bodies of arthropods. According to this argument, the true number of species could ‘be more likely in the hundreds of millions or billions’ [13]. However, we do not need to decide this controversy to hypothesize that the approximately 10,000 [11] or, respectively hundreds of thousands [12] of catalogized bacterial species/OTUs are the tips of the iceberg and that different human body sites possess extended white areas on the microbiome maps.

**Figure 1:**
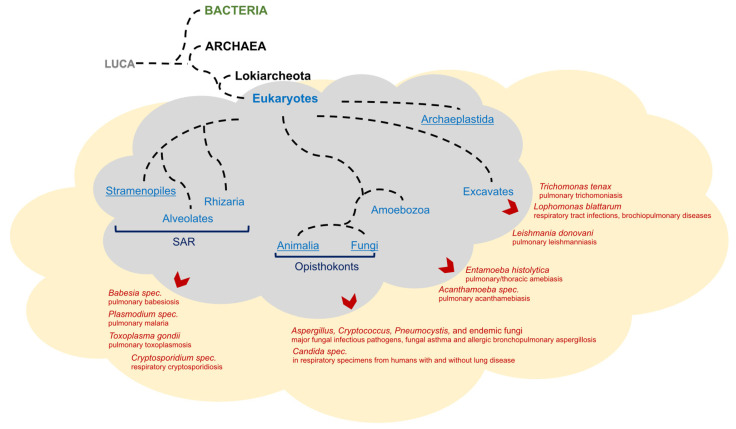
The domains of cellular life and hypothetical eukaryotic diversification. The tree of eukaryotes was adapted and simplified after Burki [18]. Pathogens from diverse eukaryotic clades contribute to human respiratory tract microbiomes and can be causally related to several diseases, e.g., apicomplexa to pulmonary babesiasis/malaria/toxoplasmosis/leishmanniasis [19] or respiratory cryptosporidiosis [20]. Multiple fungal species are associated with respiratory diseases, e.g., asthma [21], or occur in the respiratory tract [22,23]. Amoebozoa can be associated with thoracic amebiasis [24] or acanthamebiasis [19] or can be opportunistic free-living [25]. Excavates, such as *Lophomonas blattarum*, can cause respiratory tract infections and bronchopulmonary disease [26,27]. Moreover, pulmonary trichomoniasis can be related to trichomonad excavates [28]. Abbreviation: Last Universal Common Ancestor (LUCA). In underlined eukaryotic clades, multicellularity occurs, whereby Animalia exclusively contain multicellular organisms.

Considering archaea and eukaryotes, there is an ongoing debate as to whether eukaryotes and archaea represent separate domains or, more likely, eukaryotes diverged through the endosymbiosis of *alphaproteobacteria* (prospective mitochondria) through an archaeal host cell related to *Lokiarchaeota*, which as well as *Thor*-, *Odin*- and *Heimdallarchaeota* build a clade within the archaeal ‘Asgard’ superphylum [14,15]. Later in evolutionary history, the ancestors of extant eukaryotes diversified into several major clades, which contain multitudes of single-celled protozoa species as well as multicellular organisms in several subclades. The SAR supergroup includes stramenopiles, alveolates, and Rhizaria. Within the alveolates, the Apicomplexa phylum contains many known parasitic protozoa, which reside in mammalia, at least in parts of their life cycle. Unicellular and multicellular fungi are a sister kingdom of all animals (collectively called opisthokonts). Many fungi species are known parts of human microbiomes of both the alimentary and respiratory tracts. Moreover, Amoebozoa as well as several phyla of excavates can contribute to mammalian microbiomes as pathogens or harmless commensals (Figure 1).

In biomedical research, archaea are often overlooked, probably because there is still little evidence that they are associated with diseases. For example, methanogenic archaea frequently colonize the human gut, whereas their functional relevance is not well understood [16]. Similarly, whereas most unicellular eukaryotes (protozoa) are free-living organisms, of those known species that live in mammals, only a few parasites are associated with disease, whereas others are commensals. For many of the known archaeal and protozoan species being part of the mammalian microbiomes, their etiologic significance remains often unclear [17]. In order to fully characterize the diversity of human microbiomes and their relevance for health and disease, we conclusively need to expand our view with cellular life’s evolutionary history in our minds.

## 3. The Habitat: Anatomy and Physiology of the Naso- and Oropharynx

The nasopharynx is the superior part of the pharynx and extends from the base of the skull to the inferior part of the soft palate—the transition to the oropharynx [29]. The upper cervical spine is the posterior boundary [29]; anteriorly, there is the connection to the paired posterior choanaes [29,30], which are separated by the nasal septum [29].

The nasopharynx is a part of the upper airway, connecting the nasal passages to the larynx and trachea and passing the oropharynx [31]. 

**Figure 2:**
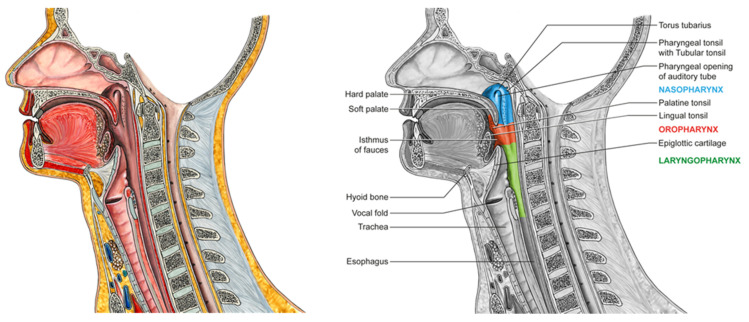
The upper respiratory tract with the naso- and oropharynx in the sagittal plane. This figure shows the division into nasal, oral and laryngeal parts of the human body. The pharynx is connected to the nasal cavity through the choanae, to the middle ear through the auditory tubes, to the oral cavity via the isthmus faucium and to the larynx via the aditus larynges [30]. This illustration was created by Jörg Pekarsky, illustrator at the Institute of Functional and Clinical Anatomy, FAU Erlangen-Nürnberg, Erlangen, Germany.

Anatomically, the oropharynx can be divided into four distinct components including the base of the tongue, the soft palate, the palatine tonsillar fossa and the pharyngeal wall [29,32]. It borders the oral cavity in the front and reaches from the soft palate to the hyoid bone [32]. Above this region, there is the nasopharynx; the hypopharynx is localized inferiorly [31,32]. The frontal connection to the oral cavity is called the isthmus faucium [30,32]. There is a palatine tonsil in each lateral wall and a lingual tonsil in the posterior one-third of the tongue [32]. These tonsils are part of the Waldeyer’s tonsillar ring, together with the pharyngeal and tubal tonsils of the nasopharynx, and the so-called side strands [30,32,33]. The structures of Waldeyer’s pharyngeal ring belong to the secondary lymphoid organs and are part of the mucosa-associated lymphoid tissue playing an important role in the human immune defense in this area [32,34]. Figure 2 presents the naso- and oropharyngeal region in detail.

## 4. The Detection of Pathogens in the Naso- and Oropharynx and Considerations Regarding Respiratory Diseases during Early Childhood

The connection of the human microbiome to the pathogenesis of diseases has gained increasing interest over many years [9]. The microbiome is defined as the total organisms, including commensalistic and parasitic cellular microorganisms, as well as viruses that inhabit the body’s surfaces [35]. 

We summarize selected studies on bacteria of the upper respiratory tract in the first part of the following subsections, followed by paragraphs on archaea and fungi. For literature searches, we applied the National Library of Medicine via PubMed to obtain manuscripts including review and research articles published in the last decade. The applied search strings were ‘microbiota’ or ‘microbiome’ and ‘oropharynx’ or ‘nasopharynx’ in combination with ‘childhood’ or ‘children’ or ‘infants’ and ‘asthma’ or ‘wheezing’ or ‘exacerbation’ or ‘respiratory tract infection’ or ‘bronchiolitis’. For the archaeome part, we used the following search strings: ‘children AND archaea’ or ‘asthma exacerbation AND archaea’ or ‘archaea nasopharynx’ or ‘archaea oropharynx’ and ‘human archaeome’ or ‘archaea AND nose AND/OR mouth’ and ‘archaea mouth nose pharynx’ and ‘archaea AND disease’. Finally, PubMed and Google Scholar were extensively searched for research papers published between 2010 and 2021 regarding the mycobiome section. Here, the search strings were as follows: ‘human mycobiome in health and disease’ and ‘human mycobiome AND asthma AND children’ or ‘fungi AND asthma AND children AND oropharyngeal/nasopharyngeal’ and ‘fungal dysbiosis AND asthma AND oral/nasal’ or ‘fungal microbiota asthma exacerbation’ and ‘fungal rhinosinusitis’ or ‘fungal microbiome’ and ‘nasopharyngeal fungal microbiome’ or ‘fungi AND nasopharynx AND oropharynx’ and ‘fungal rhinosinusitis AND nasopharynx AND oropharynx’. Furthermore, a small number of additional articles were identified from the reference lists. The search was limited to papers that were available in English and in full text.

### 4.1. The Bacterial Microbiome of the Naso- and Oropharynx in Connection with the Development of Pediatric Respiratory Diseases

The studies on the oropharynx mostly compare children with a respiratory disorder, e.g., asthma, wheezing or bronchiolitis with the aim of detecting harmful, as well as possibly protective, bacterial species [36,37,38,39,40]. 

Firstly, it was hypothesized that there is a core microbiome represented by *Prevotella*, *Streptococcus, Neisseria, Veillonella* and *Haemophilus*, as these were found both in healthy controls and in children with asthma or cystic fibrosis (CF) [36]. There are two classifications of the different microbiota: phyla and genera. In their report, Cardenas et al. described the phylum *Firmicutes* as the most common and diverse, with *Streptococcus* being the most common genus of this clade [37]. Others also described *Streptococcus* as the dominant genus in patients and healthy controls [39]. Table 1 summarizes the results of this research with samples taken from the oropharynx.

Moreover, there were some observations regarding the general composition of microbiota residing in the nasopharynx [41,42,43,44]. Some studies only included individuals with respiratory disease without considering healthy controls, whereby focusing on samples derived from the same subject at different time points or comparing different body sites [45,46,47]. 

One research group discovered five distinct biotypes in the nasopharyngeal swabs of children including *Corynebacterium*/*Dolosigranulum*, *Haemophilus*, *Moraxella*, *Staphylococcus* and *Streptococcus* [41]. Perez and colleagues analyzed nasopharyngeal samples from healthy full-term (FT) and premature (PM) infants and performed a longitudinal analysis on samples with a rhinovirus infection [42]. In the PM group, they revealed a higher within-group dissimilarity relative to FT infants and increased *Proteobacteria*, as well as decreased *Firmicutes* [42]. They identified differences in the major taxonomic groups (*Streptococcus/Moraxella/Haemophilus*) and demonstrated that these prematurity-related microbiota characteristics persisted during rhinovirus infection [42]. Thus, it can be assumed that prematurity is associated with nasopharyngeal microbiota changes beyond the neonatal stage [42], indicating that not only the age of the children but also the gestational age at birth is important in determining the composition of the microbiome [42]. An additional study focused on the examination of nasopharyngeal microbiota during the critical first year of life in a prospective cohort, capturing both viral and bacterial communities and documenting all incidents of acute respiratory infections (ARIs) [43]. Most of the infants were initially colonized with *Staphylococcus* or *Corynebacterium* before the colonization with *Aloiococcus* or *Moraxella* [43]. The research group ruled out a connection between the transient invasion of *Streptococcus, Moraxella* or *Haemophilus* and the occurrence of virus-associated ARIs [43].

A study of the nasopharyngeal microbiota in Venezuelan children with or without respiratory tract infections (RTIs) or gastrointestinal infections (GIIs) revealed differing bacterial compositions between the three different groups (healthy individuals, children with RTIs and children with GIIs) [44]. Some of them were associated with the absence of infections (also observed in developed regions) such as *Corynebacterium* [44]. However, there were also further species, which were associated with RTIs and GIIs [44]. Moreover, the group detected several bacteria that thrive in tropical humid climates; additionally, the authors assumed that reciprocal interaction occurred between the nasopharynx and the gut [44]. Thus, the region in which children live and whether there is a connection to the gut microbiota are also relevant [44] (Table 2). 

### 4.2. The Importance of the Archaeome in the Human Body

Archaea represent one of the domains of evolutionary descent and are sometimes regarded as a second prokaryotic lineage, as this was firstly assumed to be a separate prokaryotic group in 1977 [51,52,53,54]. In a review, Eme and colleagues summarize the historic classification and state important current facts concerning archaea [55]. Archaea are unicellular organisms which have self-contained DNA molecules without a cell nucleus, like bacteria (reviewed in [56]). However, they were misclassified as bacteria for a long time [56]. Archaea can be characterized by distinctive and unique features, including being the only life form that can produce biological methane by means of methanogenesis and their ability to incorporate lipids with a glycerol-1-phosphate backbone [56].

Archaea share some similarities with the aforementioned microorganisms [56]. For example, their information systems, which are relevant for DNA replication as well as gene expression, are similar to those of eukaryotes, and their metabolism is similar to that of bacteria [56]. Interestingly, extremophilic archaea frequently colonize habitats under extreme physico-chemical conditions [57,58]. However, it is also known that several species also contribute to the human commensal microbiome [59]. However, the majority of studies and publications on the human microbiome still focus on its bacterial content [60]. 

Some studies set a cut-off value (<1%) for low-abundance organisms given the concern of the risk of contamination, and these organisms are subsequently excluded from the study evaluation [61]. This could mean that the knowledge about the diversity of the human microbiome is unintentionally influenced, and the importance of the low-abundance organisms in pathophysiology or the existence of a dysbiosis may be underestimated [61]. Nevertheless, the refinement and optimization of sequencing protocols, primer pairs [62] and the increased use of techniques including next-generation sequencing (NGS) [60,62,63] help to identify additional components of the microbiome, which may not be detected through culturing techniques. In the case of archaea, the right choice of primer pairs seems to be crucial [62].

In fact, it is difficult to demonstrate or prove the exact physiological or pathological role of the archaea in the human microbiome due to the challenges of cultivation in routine microbiology laboratories and cultures [64]. Thus, there is still a large knowledge gap in this field. So far, the existence of archaea in the human microbiome has been proven in some areas of the human body, with *Methanoarchaea* seeming to dominate here [60,65,66].

Overall, representatives of the phylotypes of the archaea could be detected in several locations of the human body, i.e., skin [67], gut [68], dental plaque samples [69] and oral and nose cavity [70]. Above all, there are some members of *Methanoarchaea* which can be detected frequently in the human oral cavity, most of all *Methanobrevibacter oralis* (*M. oralis*) [71,72], as well as *M. oralis*-like species, *M. smithii* and *M. smithii-like species*, although their roles still remain unclear [60]. Other archaeal representatives, e.g., *Methanosarcina mazeii, Methanobacterium curvum* and *Methanobrevibacter oralis* can also be detected in the human oral microbiome [73]. 

Koskinen and co-workers provided the first insights into the archaeome of the human microbiome with, among other things, the archaeome of the human nose [63]. The results showed a picture of niche differentiation within the human-associated archaeal communities, and in particular, the methanogenic *Euryarchaeota* signatures were commonly detected in nasal samples, but also, skin-associated *Thaumarchaeota* were found there [63]. Moreover, the authors characterized the nasal microbiome of normosmic and hyposmic volunteers to analyze the relationship between the nasal microbiome and olfactory function in individuals, where the authors observed that the microbiomes of subjects with normosmia and hyposmia had a difference in redundancy analysis and their α-diversity [74]. 

### 4.3. The Variety of the Mycobiome

There are unicellular and multicellular species which build the large eukaryotic kingdom of fungi. With its huge diversity of 1.5 to 5 million estimated species, they have an extensive impact on the Earth’s ecosystems, also due to their functions and versatility, e.g., in metabolism, morphogenesis and ecologies [75]. In cladistics, fungi have commonly subdivided into eight phyla, twelve subphyla and several classes [76]. Even though the mycobiome only represents a small part of the human microbiome, it has a wide impact on human health. 

Interestingly, only members of four lineages, *Zycomycota, Entomophthorales, Ascomycota* and *Basidiomycota*, are able to infect humans, while there is a full lineage, *Chytridiomycota*, which is not capable of causing human infections [77]. *Cryptococcus* spp., *Malassezia* spp and *Trichosporon asahii*, members of *Basidiomycota*, which can be found within human skin microbiota, can be responsible for opportunistic infections, particularly in immunosuppressed patients [77]. *C. albicans* can also cause invasive infections in immunosuppressed patients [77]. *Aspergillus* spp. *(Ascomycota)*, particularly *A. fumigatus*, is responsible for infecting human airways [77], and its colonization may be associated with an increased risk of an invasive aspergillosis in patients [78]. 

Recently, deep-sequencing techniques have more clearly highlighted the importance of microorganisms such as fungi and archaea to the microbiome, as many of them do not grow well in vitro [79]. It turns out that the presence of specific fungal populations in some body regions is associated with certain pathologies [79]. 

Fungi are known as pathological factors for atopic diseases, in particular in asthma. In this context, there are current studies that describe the association between asthma and an altered intestinal mycobiome [80,81]. 

Ward et al. characterized samples of infants over their first month of life by their microbial colonization at different body sites including the oral site, as well as their mothers’ vaginal mycobiomes, to explore whether mode of delivery causes a difference in colonization [82]. Overall, the infant mycobiome seems to be dominated by only a few taxa, especially by representatives of the Candida species, including *C. parapsilosis*, *C. tropicalis*, *S. cerevisiae*, *C. orthopsilosis*, *C. albicans*, and *Cladosporium velox* within the infant oral mycobiome but with high intraindividual variability [82]. 

For a long time, it was assumed that representatives of the Candida species dominate the oral microbiome [83]. Ghannoum and co-workers described the oral mycobiome in healthy individuals. The authors reported a total of 101 different fungal species, which showed the omnipresence of these microorganisms [83]. The most common genera of the oral fungal microbiome were represented by the Candida species and their suborders (75%) [83]. Other isolated quantities, e.g., *Cladosporium* (65%), *Aspergillus* (35%), *Fusarium* (30%) and *Cryptococcus* were also the most commonly present microorganisms [83]. Next to the aforementioned phyla, additional studies detected the presence of *Malassezia* [84], *Eurotium*, *Penicillinum* or *Pneumocystis* [85] in the respiratory airways of healthy individuals. Table 3 summarizes these important articles in the context of atopic disorders.

## 5. Clinical Translation of the Findings Regarding the Microbiome

### 5.1. The Bacterial Microbiome in Wheezing Children

Wheezing is a common symptom in early childhood [90]. The combination of additional risk factors leads to the persistence of asthma symptoms in later years [90]. 

Pathogens are more common in infantile wheezers than in healthy controls, e.g., *Haemophilus* spp. and *Staphylococcus* spp. [37]. In contrast, there were other operational taxonomic units (OTUs) less common in this population than in healthy controls, i.e., *Veillonella* spp. [37]. A further study reported no significant differences in bacterial diversity between wheezers and healthy controls, but the authors identified an association in the kindergarten and preschool children where an increased bacterial diversity was present in the wheezing group [38]. 

In a further report of a 24-month follow-up trial, an increase in the abundance of *Neisseria* was more common in children aged 9 to 24 months who developed wheeze, whereas children without wheezing had a higher abundance of *Granulicatella* between 9 and 12 months and of *Prevotella* after 18 months of age [40].

In contrast, Rosas-Salazar and co-workers reported that an increased abundance of *Lactobacillus* during respiratory syncytial virus (RSV)-associated acute respiratory infection (ARI) in infancy was related with a reduced risk of childhood wheezing illnesses at the age of two years, possibly indicating a protective factor [47].

### 5.2. Bronchiolitis in Children and the Relation to Bacterial Microbiota in the Upper Respiratory Tract

Bronchiolitis—inflammation of the bronchioles—is a common lower respiratory infection in infancy and is most frequently caused by viruses [91]. Some of the studies analyzed the interactions between the different actors in this region in connection with the development of bronchiolitis [45,46].

Dumas et al. identified three bronchiolitis profiles by analyzing the nasopharyngeal samples [45]. They discovered that one of the three detected profiles had a significantly increased risk of developing recurrent wheezing compared to the other two profiles [45]. This group was clinically characterized by a history of breathing problems, eczema during infancy and non-RSV (mostly rhinovirus) infection and showed a *Haemophilus*-dominant or *Moraxella*-dominant microbiota profile [45]. Another research paper compared nasopharyngeal aspirate samples with nasal swabs in connection with infants hospitalized for bronchiolitis [46]. The investigators described an association between *Haemophilus*-dominant nasopharyngeal microbiota and the increased severity of bronchiolitis in the aspirates that was replicated when analyzing the microbiota acquired using nasal swabs [46]. In addition, the previously detected association of *Moraxella*-dominant nasopharyngeal microbiota with the protective function against the need for intensive care was also replicated when analyzing the nasal swabs [46]. 

### 5.3. Findings in the Microbiome of Nasopharynx in Relation to the Development of Childhood Asthma 

The Global Initiative for Asthma (GINA) guidelines describe asthma as a heterogenous disease, which is characterized by chronic airway inflammation [92,93]. Per the definition, there is a medical history of respiratory symptoms such as wheeze, shortness of breath, chest tightness and cough, varying over time and in intensity [92,93]. These symptoms are accompanied by variable expiratory airflow limitation with the possibility of developing persistence over the course of the disease [92,93]. 

Tang et al. followed developmental patterns in the nasopharyngeal microbiome during infancy by taking several samples at different time points during routine visits, as well as during episodes of respiratory illness [49]. The result was the identification of four developmental trajectories of early-life microbiota compositions, as well as a description of predominant bacteria during respiratory illness [49]. The research group correlated these with the presence of asthma at ages 6, 8, 11, 13 and 18 and found an association between *Staphylococcus*-dominated microbiota in the first six months of life and an increased risk of recurrent wheezing at age three and asthma that persisted throughout childhood [49]. This was also associated with the early onset of allergic sensitization [49]. During wheezing illnesses, the detection of rhinoviruses and the predominance of *Moraxella* were associated with asthma that persisted throughout later childhood [49]. Another paper on this topic concluded that early asymptomatic *Streptococcus* colonization could be a strong predictor of asthma and that the nasopharyngeal microbiome in general could be a determinant in the spread of infection to the lower airways [43].

Some fungal species can often be studied isolated in the sputum of people with chronic respiratory diseases such as asthma [86]. These fungi take advantage of a weakened immune system and colonize the respiratory tract, which can cause mycosis in the respective individual. This often precedes the sensitization and exacerbation of asthma symptoms [86]. Patients with severe asthma presented more fungal colonization in the lower airway tract (*Aspergillus fumigatus)*, whereas healthy subjects had a lower fungal load, such as *Malassezia* [94]. The exposure to fungal genera in early life is generally associated with the exacerbation of asthma in children [95,96,97]. Van Woerden et al. evaluated the difference between the mycobiome of asthma patients and healthy controls from London, the United Kingdom, and discovered 136 fungal species in total with a higher percentage of *Eremothecium sinecaudum* and *Systenostrema alba* in healthy patients [87]. In particular, *Psathyrella candolleana, Termitomyces clypeatus* and *Malassezia pachydermatis* were significantly elevated in the sputum of the asthmatic cohort. Furthermore, *M. pachydermatis* was detected in asthmatic patients, but not in the control cohort [87]. 

### 5.4. Association of Allergy in Childhood with Microbial Colonization of the Nasopharynx

Another relevant risk factor for asthma is atopic sensitization, in children as well as in adults [98]. This is therefore also a subject of interest in connection with the microbiome of the respiratory tract in early life [50].

One research paper concentrated on the possible connection between microbiota in the nasopharynx and the development of allergies in childhood [50]. Firstly, the group found that viral pathogens were involved in over 80% of infectious events [50]. In addition, they discovered a shift in the nasopharyngeal microbiome toward dominance by a small range of pathogenic bacterial genera—even before the viral pathogens could be detected and before acute symptoms occurred [50]. The conclusion was that the colonization of pathogenic bacteria (=illness-associated) coupled with allergic sensitization is associated with persistent wheezing in school-aged children [50]. The same bacterial genera were associated with transient wheezing in non-sensitized children that disappeared after the age of three years [50]. 

Interestingly, in terms of the human skin fungiome, there also seems to be an association between *Malassezia*-IgE binding allergens and atopic conditions such as atopic dermatitis [99]. 

### 5.5. Archaea as a Human Pathogenic Factor in Periodontal Disease

It has long been assumed in the literature that there were no human-pathogenic archaea [59,100]. By now, there are some reports suggesting a correlation between the increased presence of *Methanoarchaea*, e.g., *M. oralis* in the human oral cavity, and the appearance of periodontal disease [64,101,102,103]. 

Interestingly, there is some evidence for the existence of thermo-plasma-like organisms, which also belong to *Euryarchaeota* in the oral cavity, whose occurrence could be associated with cases of apical periodontitis [104].

Horz and Connrads discuss the cross-feeding behavior or syntrophic growth and consequently close interaction between methanogens and certain bacterial species in nature with regard to substrate utilization [64]. They thus raise the hypothesis of whether this behavior also occurs in the oral cavity, i.e., whether methanogens are involved in the growth of fermenting bacteria and thus possibly could act as opportunistic pathogens themselves [64]. Supportive of this hypothesis is a shotgun metagenomic study by Dabdoub, which shows that bacterial fermentation and the archaeal methanogenesis genes within the subgingival plaque show a strongly positive correlation [71]. Nevertheless, there is still no direct evaluation or clear evidence for a coaggregation of archaea and bacteria in subgingival communities [69]. 

### 5.6. Chronic Rhinosinusitis in Connection with the Archaeome and Fungiome 

A cross-sectional study claims to be the largest investigation of human respiratory archaea to date, which provides the first insights into the prevalence, diversity and frequency of archaea in human sinuses [105]. Here, swab samples were taken from the nasal passages of 60 subjects with and without chronic rhinosinusitis (CRS), amplified by using a nested PCR approach and the occurrence of bacterial and archaeal strains were assessed [105]. A total of 16 archaea amplicon sequence variants of the strains *Euryarchaeota* and *Thaumarchaeota* were identified [105]. Sequence variants (SVs) of archaeal origin could be detected in 7 of 60 subjects, while bacterial SVs were detected in all patients [105]. As expected, bacteria were found in the samples much more frequently than archaea [105]. Due to this significantly lower proportion of archaea within the human sinuses, it seemed unlikely to Wagner Mackenzie and colleagues that the archaeal portion of the sinus microbiota has a significant impact on, or is influenced by, the disease status of the patients with chronic sinusitis [105]. An association of CRS with the bacterial component of the microbiome presumably influences the medical condition, which seems much more likely [105]. 

While *Aspergillus*, *Bipolaris* and *Curvularia* (dematiaceous fungi) play the most important role in allergic fungal rhinosinusitis, *Alternaria* and *Cladosporium* species, in particular, are associated with chronic rhinosinusitis with nasal polyps [106]. Furthermore, due to improvements in the fungal detection techniques and protocols, there is the opportunity to detect fungal species within patients with CRS [88]. Hoggard and colleagues analyzed the sino-nasal mycobiome of patients with CRS and controls using mucosal swab samples [89]. Here, all identified fungi were *Basidiomycota* or *Ascomyota*. Furthermore, *Malassezia* species were predominant and showed a positive correlation with bacteria, including Haemophilus or Corynebacterium, as well as a negative correlation with other fungal species [89]. 

## 6. Future Outlook

There are some future considerations that can be drawn from the reviewed research papers. First of all, this information can be used to get a better understanding of the pathogenesis of different respiratory diseases in childhood. One of the reports hypothesized that the nasopharyngeal microbiota may play an important role in modulating airway inflammatory and immune responses in the vulnerable group of premature children, for example [42]. 

There is still much more investigation required to fully understand the significance of the archaeome in the human microbiome, for example, the correlation between bacteria and certain *Methanoarchaea* in terms of their cross-feeding behavior and syntrophic growth, but also in terms of their physiological and pathological function. New technology and methods of cultivation may be necessary to answer all these questions.

For research on the human mycobiome, one of the main issues in searching for information on its influence on the development of asthma and atopic diseases in early life is that this information is scarce for the naso- and oropharynx and is focused on the human gut mycobiome [107]. In addition, there are more challenges to studying the airway mycobiome due to the low fungal biomass in airways in comparison to the predominant bacteria [108]. Overall, further studies are needed in the field of the fungiome of the nasopharynx, especially with regard to their relevance to pediatric respiratory diseases including allergic diseases, atopic eczema and asthma.

## Figures and Tables

**Table 1:** Overview of articles focusing on the oropharyngeal microbiota of the upper respiratory tract of children suffering from asthma, wheezing, and exacerbation or respiratory infections from 2012 to 2021.

Research Article	Disease	Key Points
Boutin and colleagues (2017) Comparison of oropharyngeal microbiota from children with asthma and cystic fibrosis [36]	Asthma, cystic fibrosis	Core microbiome represented by *Prevotella, Streptococcus, Neisseria, Veillonella* and *Haemophilus*. Opportunistic pathogens such as *Pseudomonas, Staphylococcus* and *Streptococcus* increased in children with cystic fibrosis compared to healthy controls/asthmatic children.
Cardenas and colleagues (2012) Upper Airways Microbiota in Antibiotic-Naïve Wheezing and Healthy Infants from Tropics of Rural Ecuador [37]	Wheezing	Most common and diverse phylum: *Firmicutes* with *Streptococcus* being the most common genus. Pathogens are present significantly more often in cases of infantile wheeze compared to healthy controls exemplified by *Haemophilus* spp. and *Staphylococcus* spp. Other operational taxonomic units less common in cases than controls, notably *Veillonella* spp.
Cuthbertson and colleagues (2019) Viral respiratory infections and oropharyngeal microbiota in acutely wheezing children [38]	Wheezing	No significant difference in bacterial diversity between wheezers and healthy controls. Wheezing group: attendance at kindergarten and preschool associated with increased bacterial diversity. No significant effect of rhinovirus infection on bacterial community composition.
Hu and colleagues (2017) Dynamic oropharyngeal and faecal microbiota during treatment in infants hospitalized for bronchiolitis compared with age-matched healthy subjects [39]	Bronchiolitis	*Streptococcus* dominant in healthy children and patients. In patients: microbiota after treatment comparable to that before treatment.
Powell and colleagues (2019) Temporal association of development of oropharyngeal microbiota with early life wheeze in population-based birth cohort [40]	Wheezing	Significant increase in the abundance of *Neisseria* between 9 and 24 months in children who developed wheeze. Children without wheezing: significant increase in the abundance of *Granulicatella* between 9 and 12 months and of *Prevotella* after 18 months.

**Table 2:** Overview of research articles of the nasopharyngeal microbiota of the upper respiratory tract of children with asthma, exacerbation, wheezing and respiratory infections.

Research Article	Disease	Key Points
Kelly et al. (2017) The nasopharyngeal microbiota of children with respiratory infections in Botswana [41]	Respiratory infections	Categorization in distinct biotypes: *Corynebacterium/Dolosigranulum-, Haemophilus-, Moraxella-, Staphylococcus-* and *Streptococcus*-dominant. Pneumonia-associated: *Haemophilus, Staphylococcus* and *Streptococcus*. Respiratory-infection-associated: *Moraxella* and *Streptococcus*. Children with pneumonia and HIV: associated with lower relative abundance of *Dolosigranulum*.
Luna et al. (2018) The association between anterior nares and nasopharyngeal microbiota in infants hospitalized for bronchiolitis [46]	Bronchiolitis	Correlations between two sample types (nasal and nasopharyngeal (np)), especially relating to *Moraxella/Haemophilus* genera; by contrast, high abundance of *Staphylococcus* genus in nasal swabs compared to np samples. Replicated when analyzing nasal swabs microbiota: Association between *Haemophilus*-dominant np microbiota and increased severity of bronchiolitis. *Moraxella*-dominant np microbiota previously identified as protective against severe phenotype (need for intensive care).
Man et al. (2019) Loss of Microbial Topography between oral and nasopharyngeal microbiota and development of respiratory infections early in life [48]	Respiratory infections	Oral microbiota driven mostly by feeding type, followed by age, mode of delivery and season of sampling. Oral microbiota development not directly associated with RTI. Influx of oral taxa: *Neisseria lactamica, Streptococcus, Prevotella nanceiensis, Fusobacterium* and *Janthinobacterium* lividum in the np microbiota before and during RTIs, accompanied by reduced presence and abundance of *Corynebacterium, Dolosigranulum* and *Moraxella* spp.
Perez et al. (2017) Nasopharyngeal microbiome in premature infants and stability during rhinovirus-infection [42]	Rhinovirus infection	PM: higher within group dissimilarity relative to FT infants; increased *Proteobacteria* and decreased *Firmicutes.* Differences in the major taxonomic groups identified (*Streptococcus/Moraxella/Haemophilus*). Prematurity-related microbiota characteristics persisted during rhinovirus infection.
Rosas-Salazar et al. (2018) Nasopharyngeal Lactobacillus is associated with reduced risk of childhood wheezing illnesses following acute respiratory syncytial virus infection in infancy [47]	Wheezing, RSV infection	No association between overall taxonomic composition, diversity and richness of np microbiota during RSV ARI with development of subsequent wheeze. *Lactobacillus* consistently higher in infants who did not develop this outcome; association with reduced risk for childhood wheezing at age 2 years.
Tang et al. (2021) Developmental patterns in the nasopharyngeal microbiome during infancy are associated with asthma risk [49]	Asthma	*Staphylococcus*-dominant microbiota in first 6 months of life associated with increased risk of recurrent wheezing by age 3 years and asthma persisting through childhood; also associated with early-onset allergic sensitization. During wheezing illnesses: detection of rhinoviruses and predominance of *Moraxella* associated with asthma persisting throughout later childhood.
Teo et al. (2015) The infant nasopharyngeal microbiome impacts severity of lower respiratory infection and risk of asthma development [43]	Asthma, respiratory infections	Most infants initially colonized with *Staphylococcus* or *Corynebacterium* before stable colonization with *Alloiococcus* or *Moraxella.* Transient incursions of *Streptococcus, Moraxella,* and *Haemophilus* marked virus-associated ARIs. Early asymptomatic *Streptococcus* colonization: strong asthma predictor.
Teo et al. (2018) Airway microbiota dynamics uncover a critical window for interplay of pathogenic bacteria and allergy in childhood respiratory disease [50]	Allergy, respiratory disease, wheezing	>80% of infectious events involving viral pathogens, but accompanied by a shift in the np microbiome toward dominance by a small range of pathogenic bacterial genera; change precedes the detection of viral pathogens and acute symptoms. Colonization of illness-associated bacteria coupled with allergy sensitization associated with persistent wheeze in school-aged children; in contrast: same bacterial genera associated with transient wheeze resolving after 3 years in non-sensitized children.

Abbreviations: RSV = respiratory syncytial virus; RTI = respiratory tract infection; ARI = acute respiratory infection; FT = full-term; PM = premature.

**Table 3:** Overview of published research articles of the naso-/oropharyngeal mycobiome of the human upper respiratory tract.

Research Article	Disease	Key Points
Ward et al. (2018) Development of the human mycobiome over the first month of life and across body sites [82]	-	Characterization of the infant’s (oral/anal/skin) and mothers’ mycobiomes (anal/vaginal). Early infant microbiome contains few unique operational taxonomic units (OTUs)/taxa, often dominated by one taxon within intraindividual; most prevalent taxa across all body sites: *C. albicans.* Oral: Candida spp. (*C. parapsilosis, C. tropicalis, C. orthopsilosis)* and Saccharomycetes (S. *cerevisae).* Oral mycobiomes showed high intraindividual variability for beta diversity differences p.P. No clear progression towards a different/mature infant mycobiome during observation period. Oral mycobiome less diverse than skin and anal mycobiome within first month of life.
Ghannoum et al. (2010) Characterization of the oral fungal microbiome (mycobiome) in healthy individuals [83]	-	Oral rinse samples by healthy adult probands from Cleveland areas. Determination of basal fungal distribution = fungi present in min. 20% of probands → 15 genera. Candida most frequently within basal fungal distribution.
Porter et al. (2014) Airway Surface Mycosis in Chronic Th2-Associated Airway Disease [86]	CRS CRSsNP CRSwNP AFRS control cohort	Sinus lavage fluid and blood samples from sinus surgery patients, including CRS, patients +/− nasal polyps, AFRS and non-CRS/non asthmatic control patients. Filamentous fungi more commonly in probands with Th2-associated airway disease. Fungal-specific IgE assessed in non-Th2-associated and Th2-associated airway disease patients. Positive response to fungus determined by the highest fungus-specific IgE titer found more often in samples of Th2-associated disease patients (50%) than in control cohort. Th2-associated disease: *A. fumigatus, A. niger* (minority), *A. alternaria* (IgE more significantly). Control group: *A. alternaria, A. fumigatus, A. niger* → minority.
Van Woerden (2013) Differences in fungi present in induced sputum samples from asthma patients and non-atopic controls: a community-based case–control study [87]	Asthma control cohort	Total of 136 fungal species identified in induced sputum samples, 90 species more common in asthma patients, 46 in control subjects (based on total DNA reads). *Malassezia* was found in patients with asthma only. Asthma: *Psathyrella candolleana, M. pachydermatis, Termitomyces clypeatus* and *Grifola sordulenta.* Control cohort: *E. sinecaudum, Systenostrema alba, C. cladosporioides, Vanderwaltozyma polyspora.*
Cleland et al. (2014) The fungal microbiome in chronic rhinosinusitis: richness, diversity, postoperative changes and patient outcomes [88]	CRS control cohort	Sinus swabs collected intraoperatively; fungal outcomes determined through 18S ribosomal DNA fungal tag encoded FLX amplicon pyrosequencing. Total of several fungal genera detected; *Malassezia* in all patients the most abundant; detection of a fungal decrease postoperatively.
Hoggard et al. (2019) The sinonasal mycobiota in chronic rhinosinusitis and control patients [89]	CRS -CRSsNP -CRSwNP -CRSwCF Control cohort	Mucosal swab samples of middle nasal meatuses analyzed through Illumina MiSeq platform. *Malassezia* spp. ubiquitous, represented most abundant ZOTUs, distinct in patients with CF. Few fungal-bacterial/-inflammatory associations were observed; all identified fungi were from phyla *Basidiomycota or Ascomycota.* ZOTU1_Malassezia: associated with fungal/bacterial variables, e.g., *Haemophilus, Corynebacterium* and *Finegoldia* (positive correlation)*,* and *Aspergillus, Cladosporium, Davidiella* and *Leptosphaerulina* (negative correlation).

Abbreviations: p.P. = post-partum; ZOTU = Zero-radius Operational Taxonomic Unit; TEFAP = tag-encoded FLX amplicon pyrosequencing; CRS = chronic rhinosinusitis; AFRS = allergic fungal rhinosinusitis; CRSsNP = chronic rhinosinusitis without nasal polyps; CRSwNP = chronic rhinosinusitis with nasal polyps; CRSwCF = chronic rhinosinusitis with cystic fibrosis; CF = cystic fibrosis.

## Data Availability

The data presented in this study are available on request from the corresponding author.
